# Supplementary material for: Genomic variability of lipooligosaccharide biosynthesis locus and sequence type among Campylobacter jejuni isolated from patients with Guillain-Barré syndrome
Source: Microbiol Spectr. 2025 Jul 9;13(8):e00062-25. doi: 10.1128/spectrum.00062-25 (PMC12323628; doi:10.1128/spectrum.00062-25)
Supplement: Supplemental tables — Tables S1 to S4. [file spectrum.00062-25-s0001.docx]

**Supplementary Table S1: Genomic data of all GBS-associated and enteritis-associated strains.**

| ***C. jejuni***  **strains** | **SRA accession no.** | **Genome coverage (x)** | **Genome length (bp)** | **N_50_ (bp)** | **‘N’ bases in assembled genome** | **Avg. ‘N’ Bases/Genome (FASTQ)** | **Number of contigs** |
| --- | --- | --- | --- | --- | --- | --- | --- |
| BD10 | [JANGZE000000000](https://www.ncbi.nlm.nih.gov/nuccore/JANGZE000000000) | 496 | 1779839 | 129137 | 0 | 3405 | 72 |
| BD22 | [JANGZD000000000](https://www.ncbi.nlm.nih.gov/nuccore/JANGZD000000000) | 406 | 1740291 | 136300 | 0 | 2625 | 61 |
| BD27 | [JANGZC000000000](https://www.ncbi.nlm.nih.gov/nuccore/JANGZC000000000) | 526 | 1735253 | 108573 | 0 | 352 | 55 |
| BD34 | [JANGZB000000000](https://www.ncbi.nlm.nih.gov/nuccore/JANGZB000000000) | 569 | 1740562 | 129901 | 0 | 3429 | 62 |
| BD-39 | [NGUG00000000](https://www.ncbi.nlm.nih.gov/nuccore/NGUG00000000) | 393 | 1599909 | 145897 | 0 | 151809 | 35 |
| BD-67 | [NGUF00000000](https://www.ncbi.nlm.nih.gov/nuccore/NGUF00000000) | 842 | 1778638 | 129048 | 0 | 63378 | 62 |
| BD74 | [JANGZA000000000](https://www.ncbi.nlm.nih.gov/nuccore/JANGZA000000000) | 280 | 1766412 | 99184 | 0 | 1952 | 70 |
| BD-75 | [NGUI00000000](https://www.ncbi.nlm.nih.gov/nuccore/NGUI00000000) | 460 | 1651474 | 183845 | 0 | 182956 | 18 |
| BD94 | [JANGYZ000000000](https://www.ncbi.nlm.nih.gov/nuccore/JANGYZ000000000) | 529 | 1634649 | 214471 | 0 | 3479 | 24 |
| ZH1 | [JANGYW000000000](https://www.ncbi.nlm.nih.gov/nuccore/JANGYW000000000) | 384 | 1622417 | 153711 | 0 | 2097 | 48 |
| ZH2 | [JANGYV000000000](https://www.ncbi.nlm.nih.gov/nuccore/JANGYV000000000) | 347 | 1833494 | 106067 | 0 | 2355 | 58 |
| ZH6 | [JANGYU000000000](https://www.ncbi.nlm.nih.gov/nuccore/JANGYU000000000) | 316 | 1611064 | 147019 | 0 | 2043 | 45 |
| ZH7 | [JANGYT000000000](https://www.ncbi.nlm.nih.gov/nuccore/JANGYT000000000) | 275 | 1657009 | 183845 | 0 | 1950 | 46 |
| ZH8 | [JANGYS000000000](https://www.ncbi.nlm.nih.gov/nuccore/JANGYS000000000) | 212 | 1733715 | 159462 | 0 | 1631 | 36 |
| ZH9 | [JANGYR000000000](https://www.ncbi.nlm.nih.gov/nuccore/JANGYR000000000) | 383 | 1624889 | 141071 | 0 | 2492 | 38 |
| ZH10 | [JANGYQ000000000](https://www.ncbi.nlm.nih.gov/nuccore/JANGYQ000000000) | 667 | 1623975 | 153957 | 0 | 4533 | 40 |
| ZH11 | [JANGYP000000000](https://www.ncbi.nlm.nih.gov/nuccore/JANGYP000000000) | 354 | 1623507 | 153957 | 0 | 2387 | 38 |
| ZH12 | [JANGYO000000000](https://www.ncbi.nlm.nih.gov/nuccore/JANGYO000000000) | 300 | 1692469 | 152998 | 0 | 2193 | 31 |
| ZH14 | [JANGYN000000000](https://www.ncbi.nlm.nih.gov/nuccore/JANGYN000000000) | 253 | 1691756 | 151998 | 0 | 1846 | 31 |
| ZH15 | [JANGYM000000000](https://www.ncbi.nlm.nih.gov/nuccore/JANGYM000000000) | 422 | 1808808 | 129063 | 0 | 2833 | 186 |
| ZH16 | [JANGYL000000000](https://www.ncbi.nlm.nih.gov/nuccore/JANGYL000000000) | 338 | 1734955 | 176239 | 0 | 2346 | 48 |
| ZH18 | [JANGYK000000000](https://www.ncbi.nlm.nih.gov/nuccore/JANGYK000000000) | 461 | 1608634 | 118015 | 0 | 2991 | 39 |
| ZH19 | [JANGYJ000000000](https://www.ncbi.nlm.nih.gov/nuccore/JANGYJ000000000) | 382 | 1652750 | 183845 | 0 | 2543 | 30 |
| ZH20 | [JANGYI000000000](https://www.ncbi.nlm.nih.gov/nuccore/JANGYI000000000) | 352 | 1674438 | 147300 | 0 | 2488 | 80 |
| Z191005RS | [JAGIQH000000000](https://www.ncbi.nlm.nih.gov/nuccore/JAGIQH000000000) | 682 | 1628993 | 142512 | 200 | 6014 | 55 |
| Z191005SS | [JAGIQI000000000](https://www.ncbi.nlm.nih.gov/nuccore/JAGIQI000000000) | 758 | 1631208 | 153957 | 100 | 7008 | 55 |
| Z201020SS | [JAGIQK000000000](https://www.ncbi.nlm.nih.gov/nuccore/JAGIQK000000000) | 654 | 1616053 | 177426 | 110 | 6448 | 35 |
| Z201020RS | [JAGIQJ000000000](https://www.ncbi.nlm.nih.gov/nuccore/JAGIQJ000000000) | 730 | 1622648 | 183722 | 210 | 7597 | 60 |
| Z211080RS | [JANGYY000000000](https://www.ncbi.nlm.nih.gov/nuccore/JANGYY000000000) | 366 | 1619922 | 184432 | 0 | 2529 | 29 |
| Z211080SS | [JANGYX000000000](https://www.ncbi.nlm.nih.gov/nuccore/JANGYX000000000) | 392 | 1619938 | 192110 | 0 | 2569 | 28 |
| Z211097RS | [JARFMV000000000](https://www.ncbi.nlm.nih.gov/nuccore/JARFMV000000000) | 246 | 1684875 | 185341 | 0 | 1200 | 30 |
| Z211097SS | [JARFMU000000000](https://www.ncbi.nlm.nih.gov/nuccore/JARFMU000000000) | 214 | 1684581 | 185342 | 0 | 1047 | 37 |
| Z221123RS | [JARFMT000000000](https://www.ncbi.nlm.nih.gov/nuccore/JARFMT000000000) | 435 | 1618842 | 183726 | 0 | 1906 | 47 |
| Z221123SS | [JARFMS000000000](https://www.ncbi.nlm.nih.gov/nuccore/JARFMS000000000) | 345 | 1613609 | 177382 | 0 | 1564 | 28 |
| Z221130RS | [JARFMO000000000](https://www.ncbi.nlm.nih.gov/nuccore/JARFMO000000000) | 355 | 1807162 | 134085 | 0 | 1937 | 119 |
| Z221146RS | [JARFMR000000000](https://www.ncbi.nlm.nih.gov/nuccore/JARFMR000000000) | 304 | 1747967 | 175203 | 0 | 1550 | 43 |
| Z221146SS | [JARFMQ000000000](https://www.ncbi.nlm.nih.gov/nuccore/JARFMQ000000000) | 331 | 1755072 | 175203 | 0 | 1674 | 68 |
| Z227001SS | [JARFMP000000000](https://www.ncbi.nlm.nih.gov/nuccore/JARFMP000000000) | 309 | 1615219 | 177382 | 0 | 1463 | 36 |
| Z212061 | [JARFMW000000000](https://www.ncbi.nlm.nih.gov/nuccore/JARFMW000000000) | 476 | 1646209 | 153703 | 0 | 3160 | 29 |
| Z212063 | [JARFMX000000000](https://www.ncbi.nlm.nih.gov/nuccore/JARFMX000000000) | 577 | 1675409 | 159086 | 0 | 3774 | 45 |
| Z228001 | [JARFNH000000000](https://www.ncbi.nlm.nih.gov/nuccore/JARFNH000000000) | 364 | 1647711 | 146946 | 0 | 1684 | 36 |
| Z228003 | [JARFNG000000000](https://www.ncbi.nlm.nih.gov/nuccore/JARFNG000000000) | 273 | 1726101 | 157777 | 0 | 1308 | 37 |
| Z228004 | [JARFNF000000000](https://www.ncbi.nlm.nih.gov/nuccore/JARFNF000000000) | 313 | 1627776 | 158658 | 0 | 1448 | 64 |
| Z228006 | [JARFNE000000000](https://www.ncbi.nlm.nih.gov/nuccore/JARFNE000000000) | 388 | 1612242 | 287633 | 0 | 1676 | 29 |
| Z228007 | [JARFND000000000](https://www.ncbi.nlm.nih.gov/nuccore/JARFND000000000) | 380 | 1691314 | 189618 | 0 | 1779 | 36 |
| Z228010 | [JARFNC000000000](https://www.ncbi.nlm.nih.gov/nuccore/JARFNC000000000) | 367 | 1612813 | 177714 | 0 | 1627 | 25 |
| Z228012 | [JARFNB000000000](https://www.ncbi.nlm.nih.gov/nuccore/JARFNB000000000) | 181 | 1689694 | 173717 | 0 | 941 | 31 |
| Z228013 | [JARFNA000000000](https://www.ncbi.nlm.nih.gov/nuccore/JARFNA000000000) | 255 | 1663428 | 183066 | 0 | 1264 | 27 |
| Z228014 | [JARFMZ000000000](https://www.ncbi.nlm.nih.gov/nuccore/JARFMZ000000000) | 367 | 1615817 | 287659 | 0 | 1761 | 46 |
| Z228024 | [JARFMY000000000](https://www.ncbi.nlm.nih.gov/nuccore/JARFMY000000000) | 153 | 1645947 | 152566 | 0 | 748 | 45 |
| 600032 | [JAAJVI000000000](https://www.ncbi.nlm.nih.gov/nuccore/JAAJVI000000000) | 593 | 1834224 | 174893 | 0 | 30791 | 59 |
| 600034 | [JAAJVH000000000](https://www.ncbi.nlm.nih.gov/nuccore/JAAJVH000000000) | 755 | 1766375 | 52978 | 0 | 39284 | 92 |
| 600099 | [JAAJVG000000000](https://www.ncbi.nlm.nih.gov/nuccore/JAAJVG000000000) | 436 | 1661406 | 153905 | 0 | 32900 | 16 |
| 600978 | [JAAJVE000000000](https://www.ncbi.nlm.nih.gov/nuccore/JAAJVE000000000) | 293 | 1772006 | 158690 | 0 | 23648 | 44 |
| 601037 | [JAAJVD000000000](https://www.ncbi.nlm.nih.gov/nuccore/JAAJVD000000000) | 644 | 1671899 | 129032 | 0 | 46475 | 23 |
| 603180 | [JAAJVB000000000](https://www.ncbi.nlm.nih.gov/nuccore/JAAJVB000000000) | 531 | 1723063 | 103103 | 0 | 41440 | 48 |
| 603771 | [JAAJVA000000000](https://www.ncbi.nlm.nih.gov/nuccore/JAAJVA000000000) | 260 | 1669130 | 217195 | 0 | 18574 | 36 |
| 604421 | [JAAJUX000000000](https://www.ncbi.nlm.nih.gov/nuccore/JAAJUX000000000) | 403 | 1612509 | 183854 | 0 | 31156 | 20 |
| 604447 | [JAAJUW000000000](https://www.ncbi.nlm.nih.gov/nuccore/JAAJUW000000000) | 360 | 1605028 | 166133 | 0 | 25916 | 22 |
| 604313 | [JAAJUZ000000000](https://www.ncbi.nlm.nih.gov/nuccore/JAAJUZ000000000) | 391 | 1872995 | 64463 | 0 | 33362 | 83 |
| 600883 | [JAAJVF000000000](https://www.ncbi.nlm.nih.gov/nuccore/JAAJVF000000000) | 459 | 1734451 | 119457 | 0 | 35429 | 32 |
| 604349 | [JAAJUY000000000](https://www.ncbi.nlm.nih.gov/nuccore/JAAJUY000000000) | 437 | 1764238 | 120039 | 0 | 33515 | 52 |

**Supplementary Table S2: Nucleotide variation within the LOS region of GBS and enteritis-associated strains.**

| ***C. jejuni***  **strains** | **Total number of nucleotide variations/1kb** | **Number of SNP/1kb** | **Number of MNP/1kb** | **Number of Complex/1kb** |
| --- | --- | --- | --- | --- |
| BD10 | 21.019 | 15.92 | 0.51 | 4.58 |
| BD22 | 21.19 | 15.97 | 0.34 | 4.86 |
| BD27 | 20.67 | 15.63 | 0.68 | 4.35 |
| BD34 | 21.64 | 16.20 | 0.51 | 4.92 |
| BD-39 | 19.62 | 14.76 | 0.83 | 4.03 |
| BD-67 | 21.82 | 16.60 | 0.85 | 4.35 |
| BD74 | 20.38 | 15.29 | 0.57 | 4.52 |
| BD-75 | 18.02 | 14.52 | 0.53 | 2.96 |
| BD94 | 27.15 | 20.56 | 0.71 | 5.86 |
| ZH1 | 0 | 0 | 0 | 0 |
| ZH2 | 18.47 | 13.55 | 0.40 | 4.51 |
| ZH6 | 18.73 | 14.40 | 0.35 | 3.97 |
| ZH7 | 18.08 | 14.58 | 0.17 | 3.32 |
| ZH8 | 19.64 | 14.89 | 0.11 | 4.63 |
| ZH9 | 0.05 | 0 | 0 | 0.05 |
| ZH10 | 0.10 | 0 | 0 | 0.10 |
| ZH11 | 0 | 0 | 0 | 0 |
| ZH12 | 17.37 | 12.68 | 0.28 | 4.40 |
| ZH14 | 16.62 | 12.22 | 0.28 | 4.11 |
| ZH15 | 19.87 | 15.17 | 0.40 | 4.29 |
| ZH16 | 20.33 | 15.40 | 0.28 | 4.63 |
| ZH18 | 19.56 | 14.70 | 0.53 | 4.32 |
| ZH19 | 17.78 | 14.17 | 0.35 | 3.26 |
| ZH20 | 21.23 | 16.19 | 0.35 | 4.67 |
| Z191005RS | 0 | 0 | 0 | 0 |
| Z191005SS | 0.05 | 0 | 0 | 0.05 |
| Z201020SS | 21.22 | 15.35 | 0.83 | 5.04 |
| Z201020RS | 20.87 | 15.00 | 0.83 | 5.04 |
| Z211080RS | 22.94 | 18.65 | 0.45 | 3.83 |
| Z211080SS | 23.01 | 18.65 | 0.52 | 3.83 |
| Z211097RS | 19.75 | 15.73 | 0.68 | 3.33 |
| Z211097SS | 18.93 | 15.11 | 0.61 | 3.20 |
| Z221123RS | 19.62 | 14.52 | 0.53 | 4.56 |
| Z221123SS | 19.80 | 14.52 | 0.41 | 4.86 |
| Z221130RS | 28.58 | 21.63 | 0.78 | 6.16 |
| Z221146RS | 20.56 | 15.23 | 0.40 | 4.92 |
| Z221146SS | 20.73 | 15.29 | 0.45 | 4.98 |
| Z227001SS | 18.97 | 13.87 | 0.41 | 4.68 |
| Z212061 | 27.04 | 20.46 | 0.92 | 5.65 |
| Z212063 | 31.41 | 24.34 | 0.85 | 6.21 |
| Z228001 | 23.04 | 18.00 | 0.55 | 4.48 |
| Z228003 | 0 | 0 | 0 | 0 |
| Z228004 | 23.01 | 18.73 | 0.52 | 3.76 |
| Z228006 | 27.87 | 21.65 | 1.17 | 5.03 |
| Z228007 | 21.19 | 16.74 | 0.59 | 3.85 |
| Z228010 | 19.56 | 14.34 | 0.65 | 4.56 |
| Z228012 | 43.55 | 34.80 | 1.20 | 7.54 |
| Z228013 | 29.20 | 23.02 | 0.80 | 5.38 |
| Z228014 | 27.87 | 21.65 | 1.28 | 4.93 |
| Z228024 | 25.50 | 19.95 | 0.41 | 5.14 |
| 600032 | 18.30 | 13.26 | 0.81 | 4.22 |
| 600034 | 15.81 | 12.04 | 0.63 | 3.12 |
| 600099 | 22.05 | 16.32 | 0.74 | 4.98 |
| 600978 | 27.60 | 21.53 | 1.03 | 5.03 |
| 601037 | 19.07 | 14.03 | 0.85 | 4.18 |
| 603180 | 17.61 | 13.25 | 0.56 | 3.79 |
| 603771 | 25.00 | 19.17 | 1.28 | 4.54 |
| 604421 | 19.38 | 15.00 | 0.47 | 3.91 |
| 604447 | 28.63 | 22.33 | 1.14 | 5.15 |
| 604313 | 29.81 | 23.93 | 1.25 | 4.61 |
| 600883 | 25.93 | 20.05 | 1.15 | 4.51 |
| 604349 | 59.42 | 47.66 | 2.51 | 9.02 |

**Supplementary Table S3: List of phase variable genes within the LOS region of GBS and enteritis-associated strains.**

| **Group** | **Active phase variable gene** | **Gene length (bp)** | **Major phase variable genes*** | **GBS** | **ENT** |
| --- | --- | --- | --- | --- | --- |
| 1 | N-acylneuraminate cytidyltransferase | 315 | N-acylneuraminate cytidyltransferase**(neuA)** | 16 | 5 |
| 2 | Acetyltransferase | 224 | Acetyltransferase **(Cj1145)** | 1 | 2 |
| 3 | CMP-N-acetylneuraminate-beta-galactosamide- alpha-2,3-sialyltransferase | 292 | CMP-N-acetylneuraminate-beta-galactosamide- alpha-2,3-sialyltransferase **(cst-III)** | 13 | 3 |
| 4 | Beta-1,4-N-acetylgalactosaminyltransferase | 354 | Beta-1,4-N-acetylgalactosaminyltransferase **(cgtA)** | 13 | 3 |
| 5 | Hypothetical protein | 298 | Hypothetical protein **(Cj1137)** | 7 | 6 |
| 6 | Hypothetical protein | 151 |  | 2 | 0 |
| 7 | Hypothetical protein | 198 |  | 1 | 2 |
| 8 | Beta-1,3-galactosyltransferase / Beta-1,4-galactosyltransferase | 260 | Beta-1,3-galactosyltransferase / Beta-1,4-galactosyltransferase **(Cj1136/Cj1138)** | 0 | 2 |
| 9 | Beta-1,3-galactosyltransferase / Beta-1,4-galactosyltransferase | 118 |  | 11 | 0 |
| 10 | Beta-1,3-galactosyltransferase / Beta-1,4-galactosyltransferase | 303 |  | 1 | 0 |
| 11 | Beta-1,3-galactosyltransferase / Beta-1,4-galactosyltransferase | 295 |  | 5 | 1 |
| 12 | Probable poly (beta-D-mannuronate) O-acetylase | 499 | Probable poly (beta-D-mannuronate) O-acetylase **(Cj1132)** | 2 | 1 |
| 13 | Beta-1,4-galactosaminyltransferase | 256 | Beta-1,4-galactosaminyltransferase **(waaV)** | 8 | 2 |

*****Major phase variable genes were grouped based on similar gene functions.

**Supplementary Table S4: Summary data for Tract type and length of phase variations**

| **Repeat**  **Type** | **6 bp** | **7 bp** | **8 bp** | **9 bp** | **10 bp** | **11 bp** | **12 bp** | **Genic** | **Intergenic** | **Total** |
| --- | --- | --- | --- | --- | --- | --- | --- | --- | --- | --- |
| **G** | 0 | 0 | 10 | 55 | 33 | 5 | 0 | 102 | 1 | 103 (95.4%) |
| **T** | 0 | 0 | 0 | 0 | 2 | 0 | 0 | 2 | 0 | 2 (1.9%) |
| **TA** | 3 | 0 | 0 | 0 | 0 | 0 | 0 | 3 | 0 | 3 (2.8%) |

*bp = base pair
